# Supplementary material for: An in vitro model maintaining taxon-specific functional activities of the gut microbiome
Source: Nat Commun. 2019 Sep 12;10:4146. doi: 10.1038/s41467-019-12087-8 (PMC6742639; doi:10.1038/s41467-019-12087-8)
Supplement: Supplementary file 1 — Supplementary Information [file 41467_2019_12087_MOESM1_ESM.pdf]

SUPPLEMENTARY INFORMATION

An *in vitro* model maintaining taxon-specific functional activities of the gut microbiome

Li *et al.*

Supplementary Figure 1

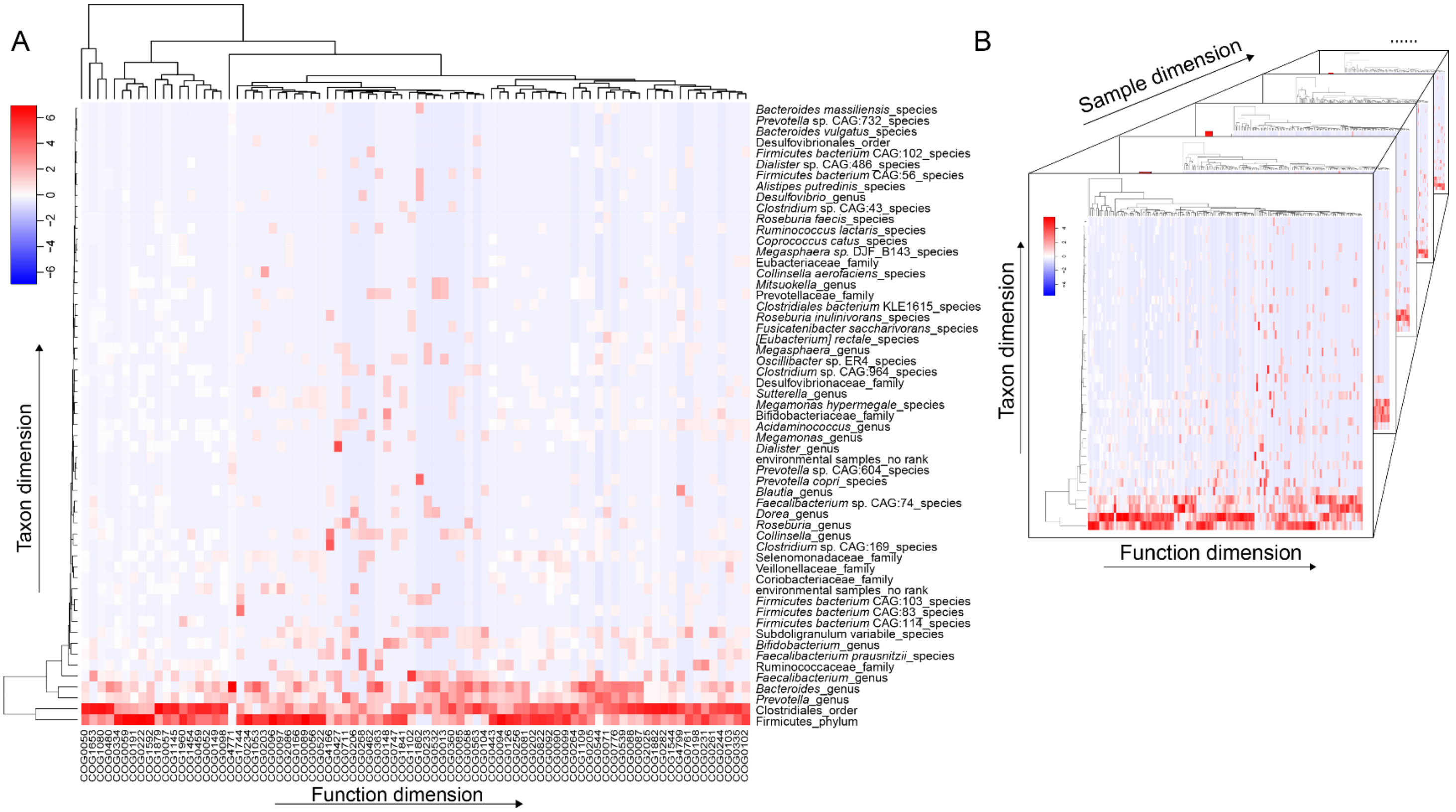

Supplementary Figure 1. Taxon-specific functional activity. Taxon-function-coupled analysis was carried out using the iMetaLab platform (http://shiny.imetalab.ca/) using the enrichment analysis module. By setting the enrichment *p* value threshold to 1, all COGs corresponding to all taxa were obtained without any filtering. (A) An overview of taxon-specific functional activity distribution in the *in vitro* validation dataset (three individual gut microbiomes inoculated 0-48 hrs in MiPro and BCM media) by selecting the “combined all samples”. (B) By selecting individual sample labels, the taxon-specific functional activity in each sample was obtained, generating a three-dimensional dataset (sample-taxon-function) for between-sample comparisons.

Supplementary Figure 2

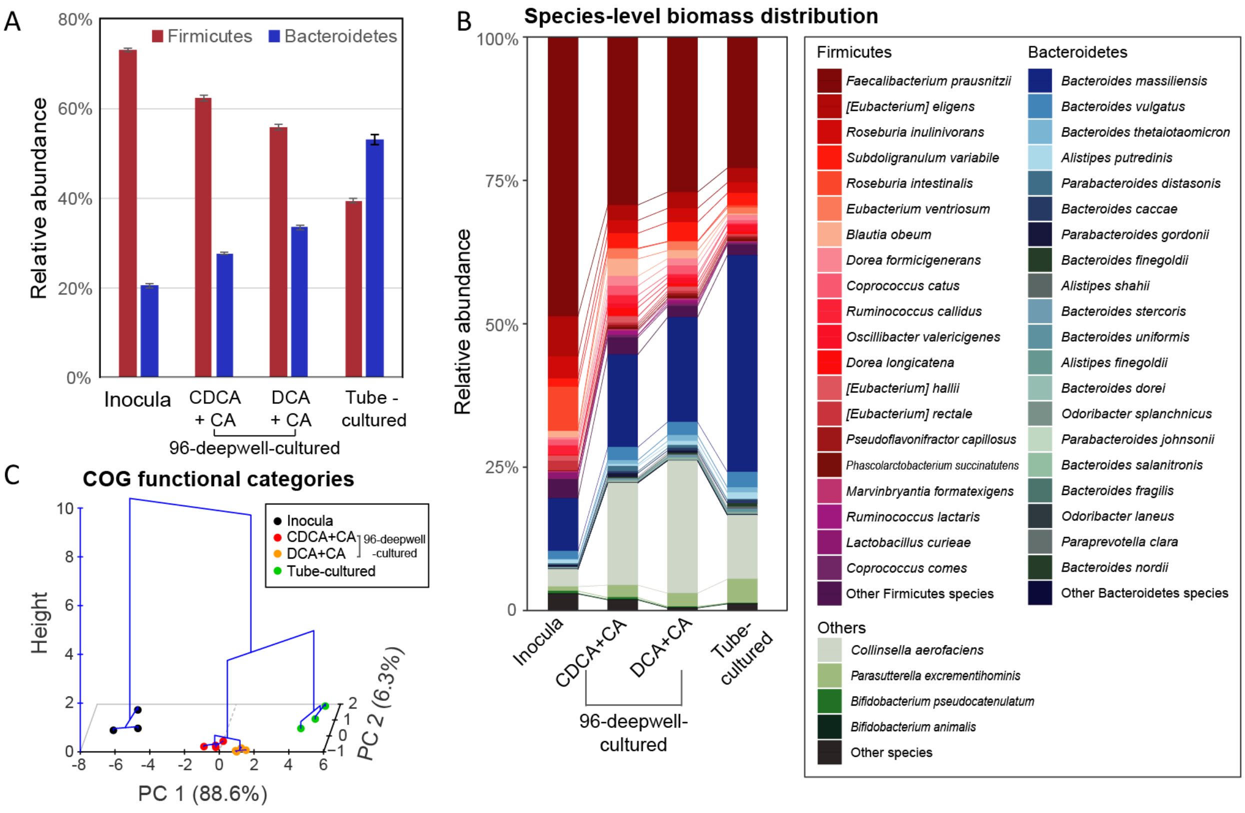

Supplementary Figure 2. Pre-test for selecting optimal bile salts composition and culture condition of the gut microbiome. (A) Pre-experiment showing the relative abundances of Firmicutes and Bacteroidetes in the inocula (0 hr baseline sample), 96-deep well cultured microbiome with the presence of primary bile salts (CDCA + CA) or commercialized bile salts mixture (DCA + CA), as well as tube-cultured microbiome with the presence of primary bile salts. Error bars represent standard deviations. (B) Compositional bar chart showing species-level biomass distribution after 24 hr culturing under the three treatments. (C) PCA scores plot with hierarchical clustering based on abundances of COG functional categories of the microbiome sample cultured under the three treatments. Samples were analyzed on an Orbitrap XL following a 6 hr gradient. 24,005 peptide sequences corresponding to 6,301 protein groups were identified with an average MS/MS identification rate of 17.6% ±2.6% (mean ± SD).

Supplementary Figure 3

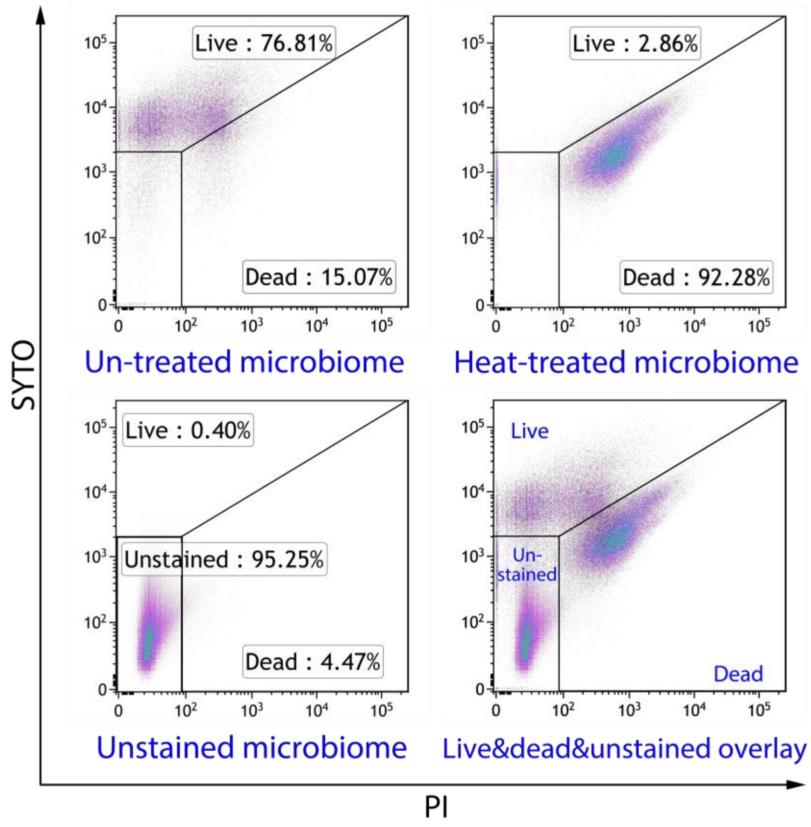

Supplementary Figure 3. Gating of live, dead and unstained bacteria according to stained gut microbiome cells, stained and heat-treated microbiome cells, and unstained microbiome.

Supplementary Figure 4

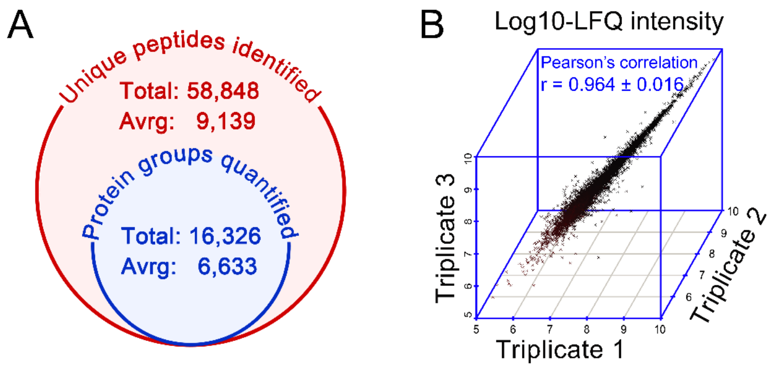

Supplementary Figure 4. Metaproteomic data quality. (A) Venn diagram showing identification efficiency of LC-MS/MS. (B) 3D scatter plot showing metaproteomic data reproducibility of technical triplicates.

Supplementary Figure 5

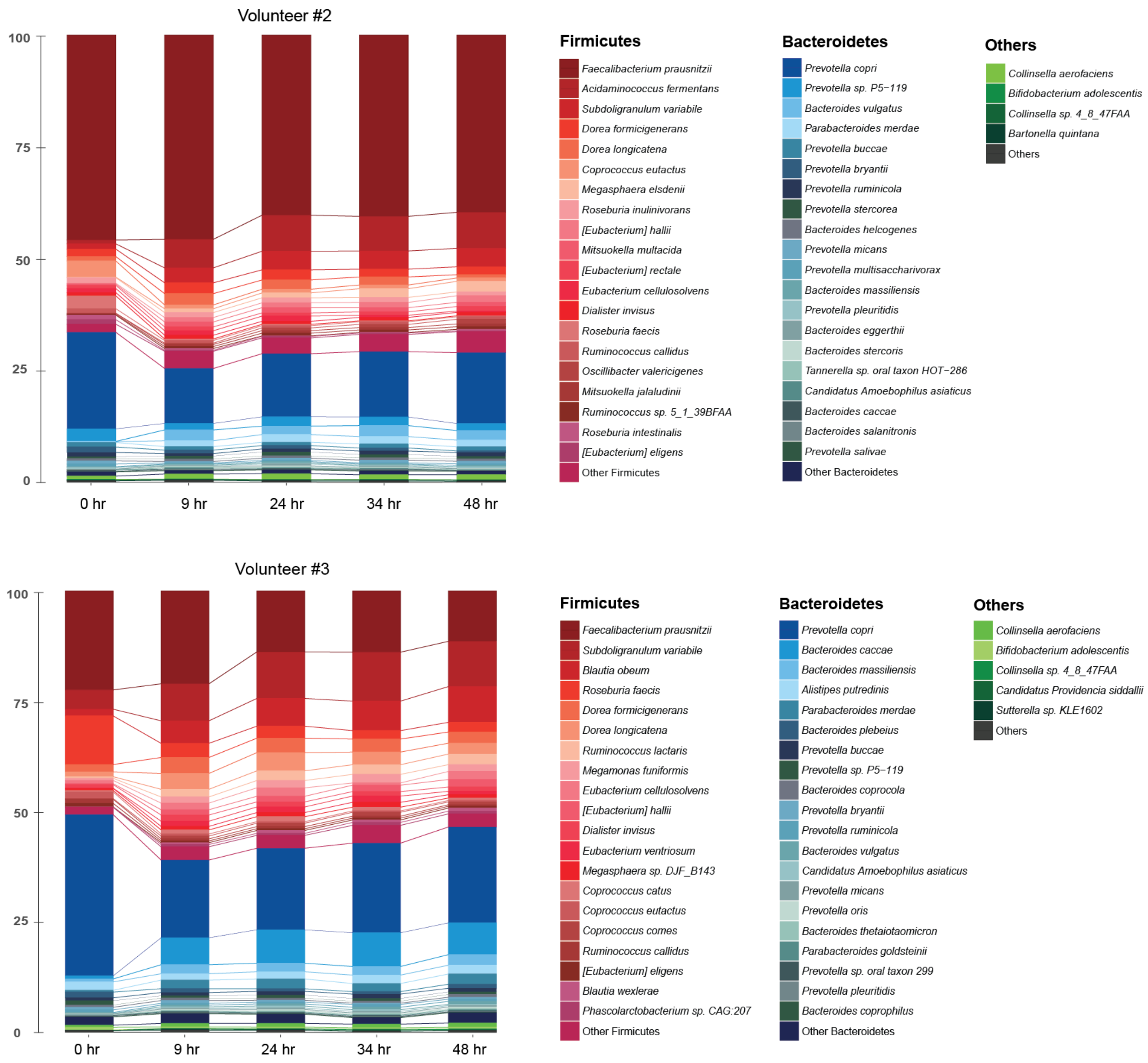

Supplementary Figure 5. Compositional bar chart showing species-level biomass distribution over time in the cultured microbiome of volunteers V2 and V3.

Supplementary Figure 6

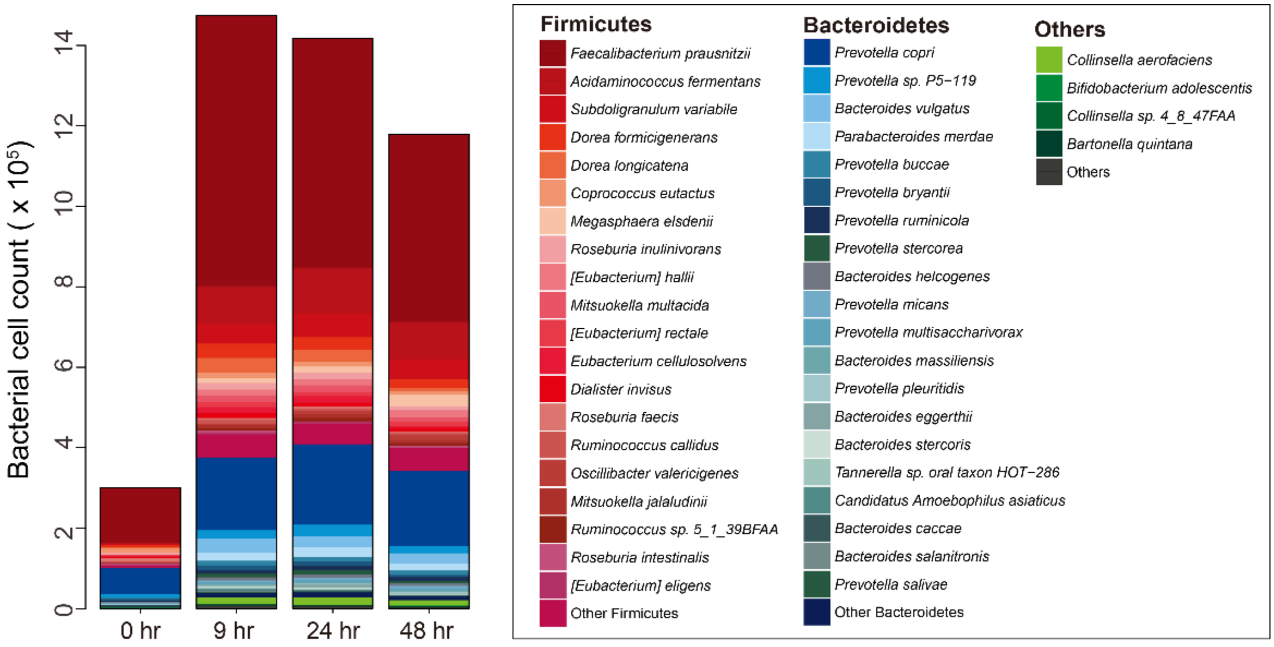

Supplementary Figure 6. Compositional bar chart of species-level biomass normalized to cell counts according to the flow-cytometry results (V2).

Supplementary Figure 7

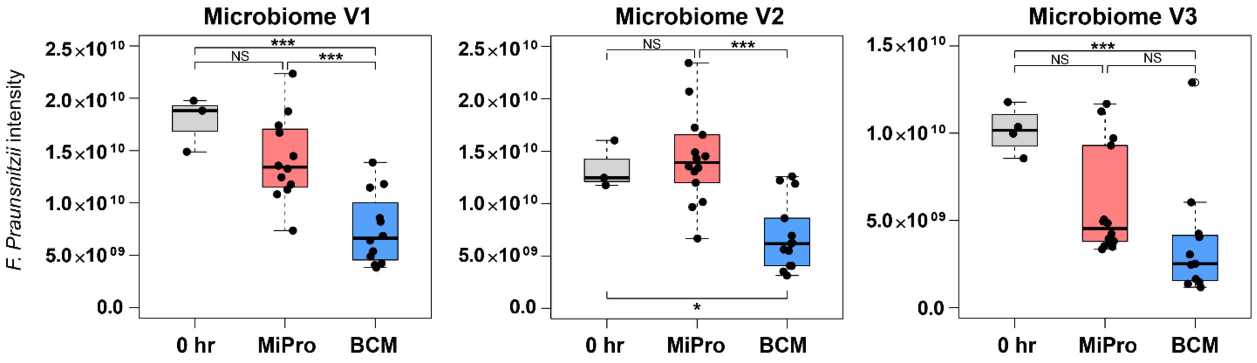

Supplementary Figure 7. Comparison of *Faecalibacterium Praunsnitzii* biomass change in the MiPro- and BCM-cultured microbiomes. Statistical significance was evaluated by Tukey's multiple comparison test and indicated as *p<0.05, ** p<0.005 and *** p<0.0005 . Box spans interquartile range (25th to 75th percentile), and line within box denotes median. Whiskers represent min to max values). Underlying data are provided in the Source Data file.

Supplementary Figure 8

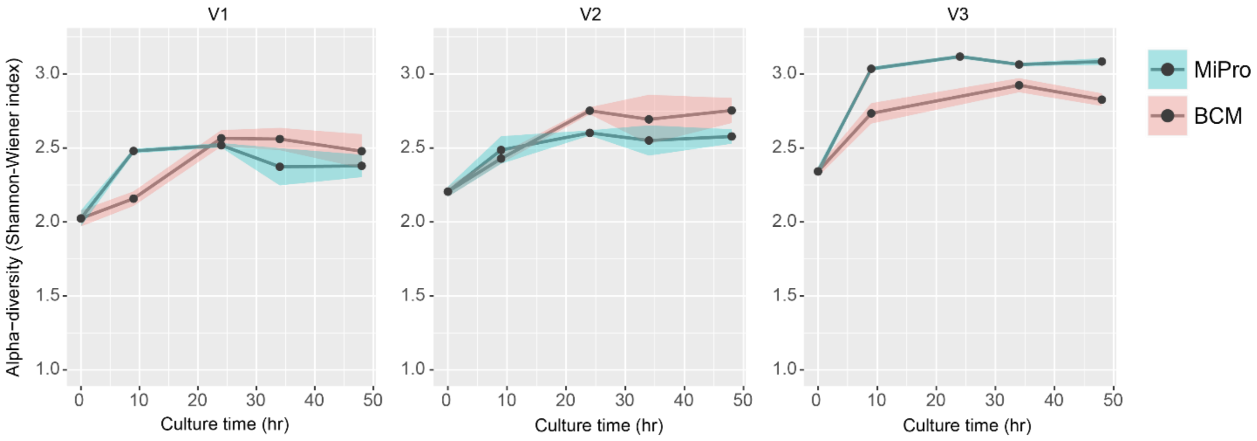

Supplementary Figure 8. Shannon-Weiner index suggesting well-maintained alpha-diversity of the microbiomes cultured from volunteers V1-3 over 48 hr. Underlying data are provided in the Source Data file.

Supplementary Figure 9

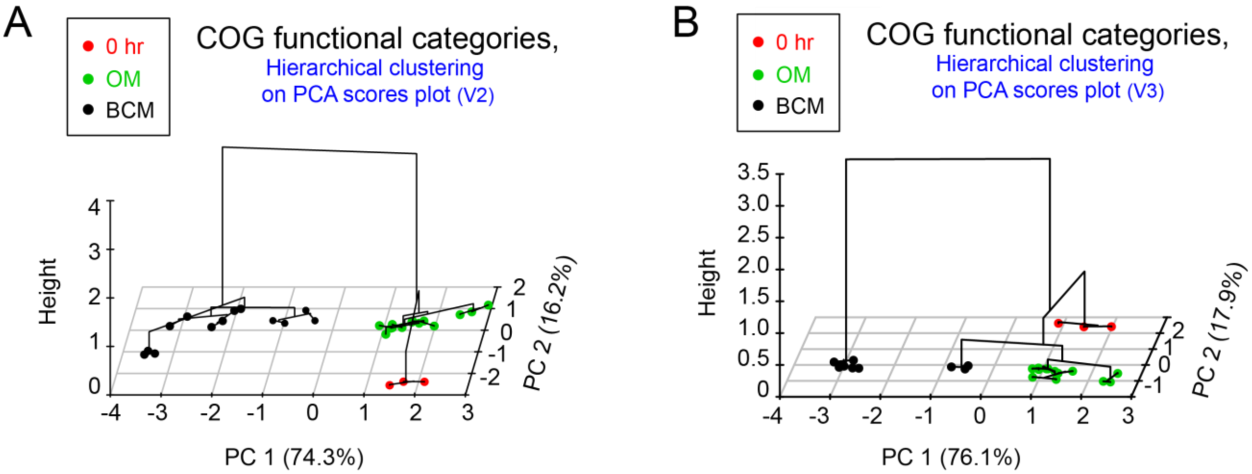

Supplementary Figure 9. PCA scores plot with hierarchical clustering based on COG functional categories of microbiome proteins from (A) volunteer V2 and (B) volunteer V3. A general discrimination of 16.2%-17.9% on the PC2 axis was contributed by culture difference, whereas a larger separation on PC1 axis (74.3%-76.1%) was induced by culture in BCM medium, suggesting better functional maintenance of microbiome cultured in MiPro medium.

Supplementary Figure 10

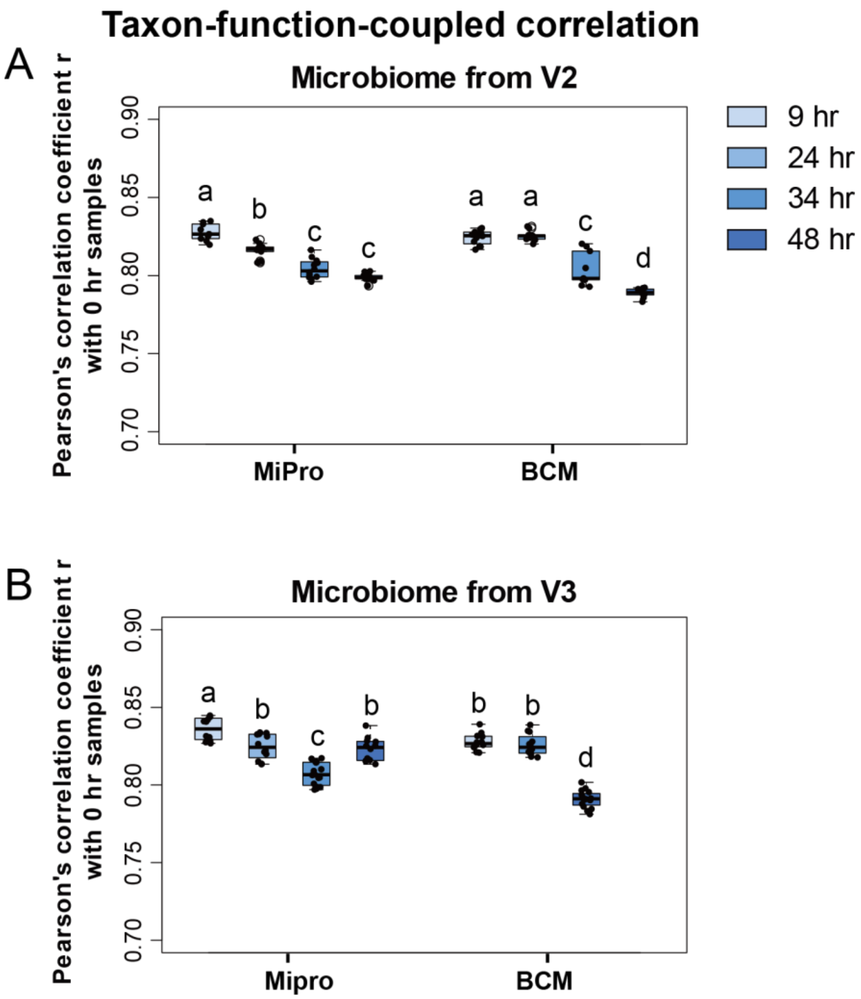

Supplementary Figure 10. Taxon-function-coupled profile in comparison with 0 hr baseline samples. (A-B) Pearson's correlation coefficient r of taxon-specific functional profiles between cultured and the inocula microbiomes of volunteers V2 (A) and V3 (B). Different letters indicate significant differences ($p < 0.05$) as determined by Tukey-b test. Box spans interquartile range (25th to 75th percentile), and line within box denotes median. Whiskers represent min to max values. Underlying data are provided in the Source Data file.

Supplementary Figure 11

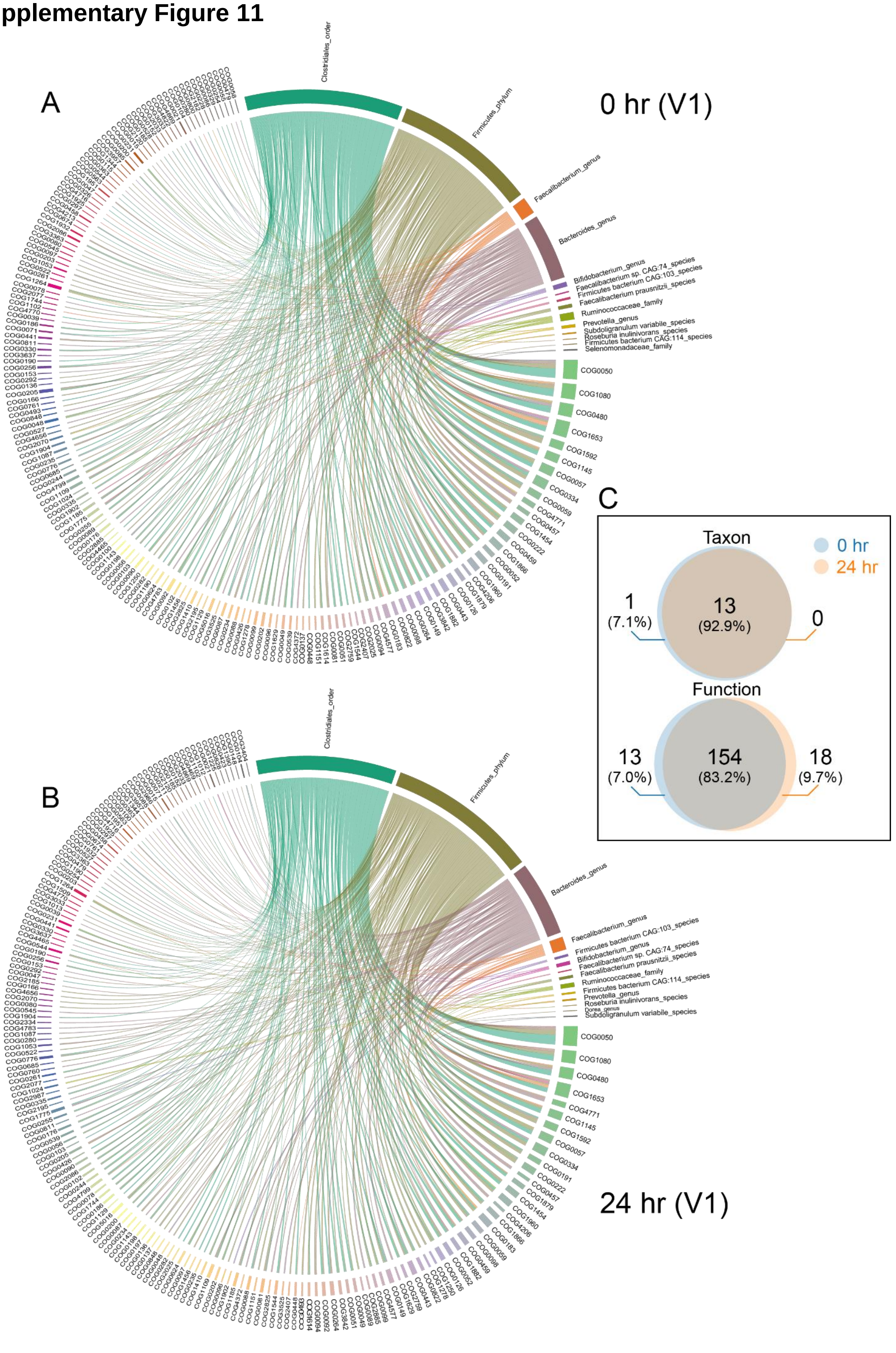

Supplementary Figure 11. Comparison of top 300 enriched taxa-function correlations, between (A) 0 hr baseline sample and (B) 24 hr MiPro-cultured sample; (C) Venn diagram showing overlapped taxa and functions between the two groups.
