# Supplementary material for: An in vitro model maintaining taxon-specific functional activities of the gut microbiome
Source: Nat Commun. 2019 Sep 12;10:4146. doi: 10.1038/s41467-019-12087-8 (PMC6742639; doi:10.1038/s41467-019-12087-8)
Supplement: Supplementary file 3 — Reporting Summary [file 41467_2019_12087_MOESM3_ESM.pdf]

nature research

Corresponding author(s): Daniel Figeys

Last updated by author(s): Aug 8, 2019

Reporting Summary

Nature Research wishes to improve the reproducibility of the work that we publish. This form provides structure for consistency and transparency in reporting. For further information on Nature Research policies, see Authors & Referees and the Editorial Policy Checklist.

Statistics

For all statistical analyses, confirm that the following items are present in the figure legend, table legend, main text, or Methods section.

n/a	Confirmed	
☐	☒	The exact sample size (*n*) for each experimental group/condition, given as a discrete number and unit of measurement
☐	☒	A statement on whether measurements were taken from distinct samples or whether the same sample was measured repeatedly
☐	☒	The statistical test(s) used AND whether they are one- or two-sided<br>*Only common tests should be described solely by name; describe more complex techniques in the Methods section.*
☒	☐	A description of all covariates tested
☐	☒	A description of any assumptions or corrections, such as tests of normality and adjustment for multiple comparisons
☐	☒	A full description of the statistical parameters including central tendency (e.g. means) or other basic estimates (e.g. regression coefficient) AND variation (e.g. standard deviation) or associated estimates of uncertainty (e.g. confidence intervals)
☐	☒	For null hypothesis testing, the test statistic (e.g. *F*, *t*, *r*) with confidence intervals, effect sizes, degrees of freedom and *P* value noted<br>*Give P values as exact values whenever suitable.*
☒	☐	For Bayesian analysis, information on the choice of priors and Markov chain Monte Carlo settings
☒	☐	For hierarchical and complex designs, identification of the appropriate level for tests and full reporting of outcomes
☐	☒	Estimates of effect sizes (e.g. Cohen's *d*, Pearson's *r*), indicating how they were calculated

Our web collection on statistics for biologists contains articles on many of the points above.

Software and code

Policy information about availability of computer code

Data collection	Thermo Xcalibur 3.1.66.10 was used for LC-MS/MS data collection. Gen5 1.11 was used for OD reading. BD FACSCelesta™ software was used for flow cytometric data collection.
Data analysis	MetaLab 1.0 was used for metaproteomic data processing. Kaluza Beckman-Coulter 1.5 was used for flow cytometry data analysis. Online platform iMetaLab (imetalab.ca) was used for taxa-functions enrichment analysis and compositional bar chart. Online platform MetaboAnalyst (www.metaboanalyst.ca) was used for PLS-DA analysis and heatmap. PASW Statistics 18 was used for ANOVA and t-tests. R software version 3.4.3 was used for Bray-Curtis dissimilarity and analysis of similarities (ANOSIM) (using package 'vegan'), principal coordinates analysis (PCoA), principle component analysis (PCA), hierarchical clustering and correlation analyses.

For manuscripts utilizing custom algorithms or software that are central to the research but not yet described in published literature, software must be made available to editors/reviewers. We strongly encourage code deposition in a community repository (e.g. GitHub). See the Nature Research guidelines for submitting code & software for further information.

Data

Policy information about availability of data

All manuscripts must include a data availability statement. This statement should provide the following information, where applicable:

- Accession codes, unique identifiers, or web links for publicly available datasets
- A list of figures that have associated raw data
- A description of any restrictions on data availability

All raw data from LC-MS/MS have been deposited to the ProteomeXchange Consortium (http://www.proteomexchange.org) via the PRIDE partner repository (dataset identifiers PXD010134, PXD010135 and PXD013600).

Field-specific reporting

Please select the one below that is the best fit for your research. If you are not sure, read the appropriate sections before making your selection.

☒ Life sciences ☐ Behavioural & social sciences ☐ Ecological, evolutionary & environmental sciences

For a reference copy of the document with all sections, see nature.com/documents/nr-reporting-summary-flat.pdf

Life sciences study design

All studies must disclose on these points even when the disclosure is negative.

Sample size	Human microbiome samples were collected from 3 individuals. For each individual, samples were analyzed using LC-MS/MS at 9, 24, 34 and 48 hr of growth. A total of 90 LC-MS/MS raw files were obtained. Seven C57/BL6 mice were included for in vitro - in vivo correlation study. In vivo samples were collected at days 0, 14 and 28 of treatment. In vitro samples were collected at 0 hr and 24 hr of culturing. One of the mice didn't produce sufficient sample to perform LC-MS/MS on day 14 of the in vivo treatment. Thus, 34 LC-MS/MS raw files were obtained for analysis.
Data exclusions	All data were included.
Replication	For human microbiome culturing, each treatment (each medium, each time point) were cultured in 4 replicates. 3-4 replicates of the cultured microbiome (in each medium at each time point) were analyzed using LC-MS/MS. Cultured triplicates showed high correlation at the quantified protein groups level (Pearson's r = 0.97 ± 0.02), indicating good technical reproducibility. For the mice study, 7 mice were used for biological replicates. Technical replication wasn't performed in mice microbiome culturing due to insufficient size of inoculum that can be obtained from each mice.
Randomization	Metaproteomic sample processing and LC-MS/MS analysis were carried out in randomized order.
Blinding	The investigators were blinded to human volunteers during sample collection and experiment. The investigators were not blinded to treatment and control samples during culturing, but were blinded for metaproteomic analysis due to randomization.

Reporting for specific materials, systems and methods

We require information from authors about some types of materials, experimental systems and methods used in many studies. Here, indicate whether each material, system or method listed is relevant to your study. If you are not sure if a list item applies to your research, read the appropriate section before selecting a response.

Materials & experimental systems

n/a	Involved in the study	
☒	☐	Antibodies
☒	☐	Eukaryotic cell lines
☒	☐	Palaeontology
☐	☒	Animals and other organisms
☐	☒	Human research participants
☒	☐	Clinical data

Methods

n/a	Involved in the study	
☒	☐	ChIP-seq
☐	☒	Flow cytometry
☒	☐	MRI-based neuroimaging

Animals and other organisms

Policy information about studies involving animals; ARRIVE guidelines recommended for reporting animal research

Laboratory animals	The study involved 7-week old male inbred C57/BL6 mice.
Wild animals	The study did not involve wild animals.
Field-collected samples	The study did not involve field-collected samples.
Ethics oversight	The animal use protocol was approved by the Animal Care Committee at the University of Ottawa (# BMI 2848).

Note that full information on the approval of the study protocol must also be provided in the manuscript.

Human research participants

Policy information about studies involving human research participants

Population characteristics	Three healthy human participants (age > 18, male and female) were involved. The volunteers were selected based on the criteria

Population characteristics	of: no diagnosis of irritable bowel syndrome, Crohn's disease, ulcerative colitis or celiac disease, no diabetes (type I or type II); no antibiotic use in the last 3 months; no episode of gastroenteritis within 3 months; no pro- or pre-biotic in the last month; no laxative use in last month; no anti-diarrheal drugs in last month; and no pregnancy.
Recruitment	The volunteers were recruited based on the above-stated criteria by a research coordinator. The investigators were blinded to human volunteers to avoid potential self-selection bias.
Ethics oversight	The human stool sampling protocol was approved by the Ottawa Health Science Network Research Ethics Board at the Ottawa Hospital (# 20160585-01H). All participants signed informed consent to participate in the study.

Note that full information on the approval of the study protocol must also be provided in the manuscript.

Flow Cytometry

Plots

Confirm that:

☒ The axis labels state the marker and fluorochrome used (e.g. CD4-FITC).

☒ The axis scales are clearly visible. Include numbers along axes only for bottom left plot of group (a 'group' is an analysis of identical markers).

☒ All plots are contour plots with outliers or pseudocolor plots.

☒ A numerical value for number of cells or percentage (with statistics) is provided.

Methodology

Sample preparation	Samples were prepared using the LIVE/DEAD BacLight Kit (Thermo Fisher Scientific Cat# L7012). Briefly, according to the manufacturer's instruction, microbial cells were washed and diluted in 0.85% NaCl saline buffer. Then, the bacteria were stained with PI and SYTO for 15 minutes prior to acquisition on the flow cytometer. For maximum bacterial cell viability, all sample processing procedures, including mixing, dilution and staining steps were performed inside the anaerobic station.
Instrument	BD FACSCelesta™ multicolor cell analyzer.
Software	Data were analyzed using Kaluza Analysis Software version 1.5.
Cell population abundance	Samples were gut microbial communities other than pure cultures. Samples were washed in 0.85% NaCl saline buffer to remove non-bacterial debris. Samples were gated as shown in Supplementary Figure S1 to remove noise. Total number of FSC/SSC gated events for each test (120 s) ranged from 300,548 in the inoculum to 1,417,981 after 24 hr of culturing.
Gating strategy	Bacterial cells were gated according to size and granularity on the FSC/SSC scatter plot. Live bacterial count was done in reference to the SYTO+ PI- gate. Live bacteria and heat-treated bacteria (dead) were used for determination of the gates. Details of gating strategy have been provided in the supplementary material.

☒ Tick this box to confirm that a figure exemplifying the gating strategy is provided in the Supplementary Information.
